# Lipoprotein Lipidomics as a Frontier in Non-Alcoholic Fatty Liver Disease Biomarker Discovery

**DOI:** 10.3390/ijms25158285

**Published:** 2024-07-29

**Authors:** Luis V. Herrera-Marcos, Jose M. Arbones-Mainar, Jesús Osada

**Affiliations:** 1 Departamento de Bioquímica y Biología Molecular y Celular, Facultad de Veterinaria, Universidad de Zaragoza, E-50013 Zaragoza, Spain; l.vte.herrera@gmail.com (L.V.H.-M.); josada@unizar.es (J.O.); 2 Instituto Agroalimentario de Aragón, CITA-Universidad de Zaragoza, E-50013 Zaragoza, Spain; 3 Instituto de Investigación Sanitaria (IIS) Aragon, E-50009 Zaragoza, Spain; 4 CIBER Fisiopatología Obesidad y Nutrición (CIBERObn), Instituto Salud Carlos III, E-28029 Madrid, Spain; 5 Adipocyte and Fat Biology Laboratory (AdipoFat), Translational Research Unit, University Hospital Miguel Servet, E-50013 Zaragoza, Spain; 6 Instituto Aragonés de Ciencias de la Salud (IACS), E-50009 Zaragoza, Spain

**Keywords:** liver, lipidomic, lipoproteins, NAFLD, NASH, biomarkers

## Abstract

Non-alcoholic fatty liver disease (NAFLD) is a progressive liver disease characterized by the build-up of fat in the liver of individuals in the absence of alcohol consumption. This condition has become a burden in modern societies aggravated by the lack of appropriate predictive biomarkers (other than liver biopsy). To better understand this disease and to find appropriate biomarkers, a new technology has emerged in the last two decades with the ability to explore the unmapped role of lipids in this disease: lipidomics. This technology, based on the combination of chromatography and mass spectrometry, has been extensively used to explore the lipid metabolism of NAFLD. In this review, we aim to summarize the knowledge gained through lipidomics assays exploring tissues, plasma, and lipoproteins from individuals with NAFLD. Our goal is to identify common features and active pathways that could facilitate the finding of a reliable biomarker from this field. The most frequent observation was a variable decrease (1–9%) in polyunsaturated fatty acids in phospholipids and non-esterified fatty acids in NAFLD patients, both in plasma and liver. Additionally, a reduction in phosphatidylcholines is a common feature in the liver. Due to the scarcity of studies, further research is needed to properly detect lipoprotein, plasma, and tissue lipid signatures of NAFLD etiologies, and NAFLD subtypes, and to define the relevance of this technology in disease management strategies in the push toward personalized medicine.

## 1. Introduction

Non-alcoholic fatty liver disease (NAFLD), also dubbed MAFLD (metabolic dysfunction-associated fatty liver disease), or more recently MASLD (metabolic dysfunction-associated steatotic liver disease) [1], is a progressive liver disease characterized by the build-up of fat in the liver of individuals with little or no alcohol intake. While diagnosing NAFLD mainly involves excluding significant or excessive alcohol consumption, MAFLD and MASLD include metabolic risk factors in their diagnostic criteria [1]. The global prevalence of NAFLD stands at approximately 25%, showing a notable upward trend in recent years paralleling the rise in obesity prevalence [2,3]. NAFLD is a heterogeneous and complex disease whose first stage, simple steatosis, has the potential to evolve into severe complications such as steatohepatitis (NASH), various degrees of fibrosis, cirrhosis, and hepatocellular carcinoma (HCC) [4]. To explain this sequence, several hypotheses have been proposed, from the “two hits” suggesting that the fat accumulation makes the liver more susceptible to an additional insult, to a multiple hit interaction of factors such as the nature of hepatic lipids, insulin resistance, oxidative stress, apoptotic, or adipocytokine involvement [5]. In addition, current diagnostic methods for NAFLD are limited by their invasiveness and the sophisticated nature of imaging technologies, which are not universally available in clinical settings. Specifically, while imaging methods such as abdominal ultrasonography and computed tomography are commonly used, they have low sensitivity during the early stages of the disease and are less effective in processing large patient populations. Consequently, liver biopsy remains the gold standard for diagnosing and staging NAFLD, despite its invasiveness and the complexity of the procedure. [6,7]. These limitations underscore the urgent need for developing new, non-invasive biomarkers through lipidomic analysis.

NAFLD’s etiology is also multifactorial, encompassing various genetic, environmental, and lifestyle factors beyond energy imbalance. Genetic predispositions, including polymorphisms I148M of *PNPLA3* and E167K of *TM6SF2*, have been implicated in increasing susceptibility to NAFLD. Specifically, these polymorphisms have been shown to affect lipid synthesis and VLDL secretion, increasing the susceptibility to fat accumulation in the liver [8,9,10].

Additionally, insulin resistance (IR), often associated with obesity and metabolic syndrome, plays a pivotal role in NAFLD pathogenesis by promoting hepatic lipid accumulation [11]. Environmental factors such as exposure to environmental toxins and pollutants can also contribute to NAFLD development through mechanisms involving oxidative stress and inflammation [12]. Moreover, emerging evidence suggests a potential link between gut microbiota dysbiosis and NAFLD progression, highlighting the intricate interplay between the gut–liver axis and metabolic health [13]. Furthermore, dietary factors beyond caloric intake, such as the quality of macronutrients consumed, particularly a high intake of fructose and saturated fats, can exacerbate hepatic steatosis and inflammation [14]. Alcohol consumption, even at moderate levels, can exacerbate liver injury in individuals with NAFLD, highlighting the importance of alcohol avoidance in disease management [15]. Sedentary behavior and physical inactivity contribute significantly to NAFLD pathogenesis by promoting IR and visceral adiposity, further exacerbating metabolic dysfunction [16]. Sleep disturbances, including obstructive sleep apnea, have also been linked to NAFLD development through mechanisms involving disrupted metabolic homeostasis and hormonal regulation [17]. Psychosocial factors such as chronic stress and depression have been associated with an increased risk of NAFLD, likely through their effects on dietary behaviors, physical activity levels, and neuroendocrine pathways [18]. Additionally, certain medications, including amiodarone, methotrexate, corticosteroids, tamoxifen, and some antiretroviral drugs, may contribute to hepatic steatosis as a side effect [19].

**Figure 1:**
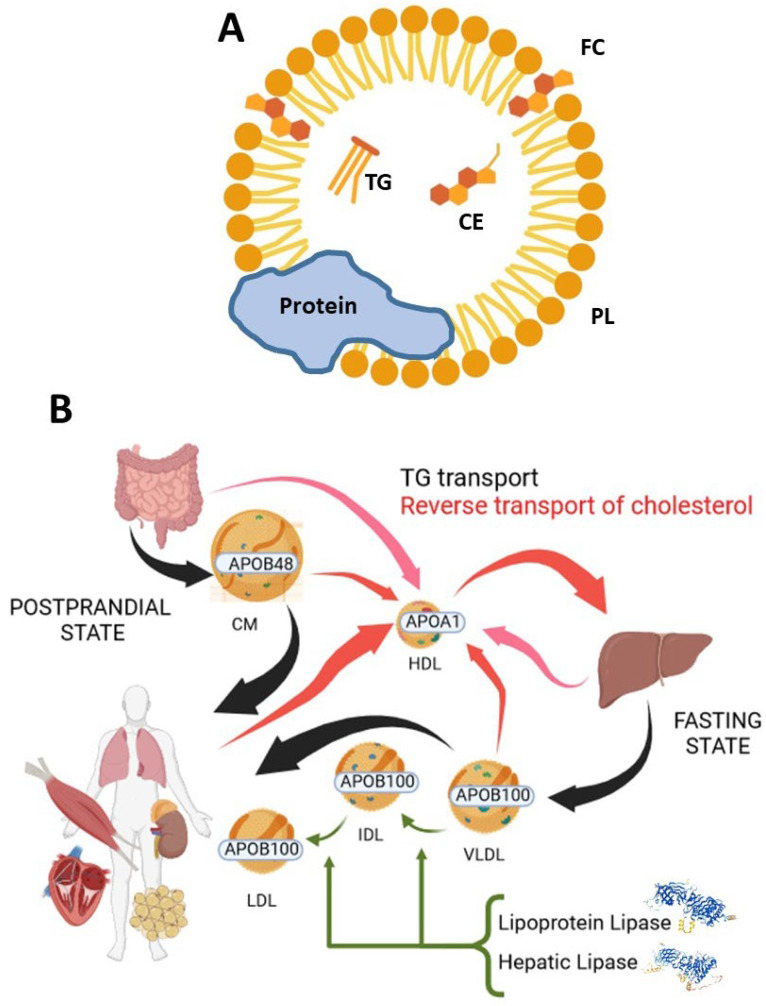
Plasma lipoproteins: (**A**) Lipoprotein biomolecular composition: CE: Cholesteryl ester; FC: free cholesterol; PL: Phospholipids; TG: Triglycerides. (**B**) Lipoprotein metabolism. Created in Biorender.com (accessed on 6 May 2024).

At the biochemical level, NAFLD manifests as a dysregulation in liver lipid metabolism, where processes favoring lipid acquisition and synthesis outweigh those facilitating lipid utilization and secretion into bile or the bloodstream [20]. Notably, lipoprotein metabolism plays a crucial role in mediating the later mechanism. Lipoproteins are complex particles formed by a combination of lipids and proteins (mainly members of the apolipoprotein family). The former are arranged as follows: cholesteryl esters (CEs) and triglycerides (TGs) as a central core and free cholesterol (FC) and phospholipids (PLs) surrounding them and forming a monolayer (Figure 1A). Plasma lipoproteins can be divided into several classes with different compositions and functions: chylomicrons (CMs), very low-density lipoproteins (VLDLs), low-density lipoproteins (LDLs), intermediate-density lipoproteins (IDLs), high-density lipoproteins (HDLs), and lipoprotein (a) (Lp (a)), and their mechanism and interaction determine the liver’s fate. Roughly, chylomicrons are produced in the intestine and contain apolipoprotein (APO) B-48, their mission is to provide dietary TGs to all tissues in the postprandial period and, finally, their remnants are taken up by the liver. VLDLs, produced by the liver, contain APOB-100 and supply fatty acids to the rest of the tissues in the fasting state. LDLs and IDLs are the subsequent products resulting from VLDL metabolism by lipoprotein lipase and hepatic lipase [21]. HDLs are produced in the liver (and intestine to a certain extent), and they are in charge of the reverse transport of cholesterol after interacting with tissues, CMs, VLDLs, LDLs, and IDLs [22] (Figure 1B). Lp(a) role has proven elusive, its most likely function would be wound healing, but also high levels of Lp(a) have been linked to cardiovascular disease [23]. Regardless of this complex mechanism, the survey of the plasma lipidome in clinical practice is based on the exploration of only two lipid subclasses in the bloodstream with scarce consideration of lipoprotein metabolism. The so-called lipid profile is circumscribed to TGs and cholesterol, differentiating in the latter between total cholesterol, the cholesterol carried by HDL (HDL-C), and cholesterol in low-density lipoprotein (LDL-C) without further consideration of other lipids in the different lipoproteins [24]. Considering the important role of the liver as a source of VLDLs and HDLs (Figure 1B) and being the fate of HDLs exerting reverse cholesterol transport, the imbalance imposed by NAFLD pathology to this organ may be reflected on its secreted lipoproteins and characteristic of their lipidomes. An approach that undoubtedly requires further attention.

To explore the complexity of lipid metabolism and lipoproteins, a promising tool has emerged in the last decades: lipidomics. This technology holds much promise in NAFLD research as we still strive for earlier identification of disease, better staging of the diversity of the process, and improving disease management strategies in the push toward personalized medicine. Various complementary approaches exist for lipidome analysis, including nuclear magnetic resonance (NMR), shotgun lipidomics (which involves directly infusing samples into a mass spectrometer with multiple scan modes throughput), targeted lipidomics (utilizing liquid chromatography with internal standards in conjunction with mass spectrometry to accurately quantify hundreds of lipid species), untargeted lipidomics (similar to targeted lipidomics but many of the studied lipids may be unidentified), and spatial lipidomics (such as matrix-assisted laser desorption/ionization-mass spectrometric, MALDI-MSI, imaging, which enables the investigation of lipid distribution within tissues) [24,25]. These approaches have been used to characterize the plasma, tissue, cell, and lipoprotein lipidome in order to investigate the relationship between lipid metabolism and NAFLD. Here we highlight how these studies have contributed to our understanding of the disease process and the potential for future contributions.

**Figure 2:**
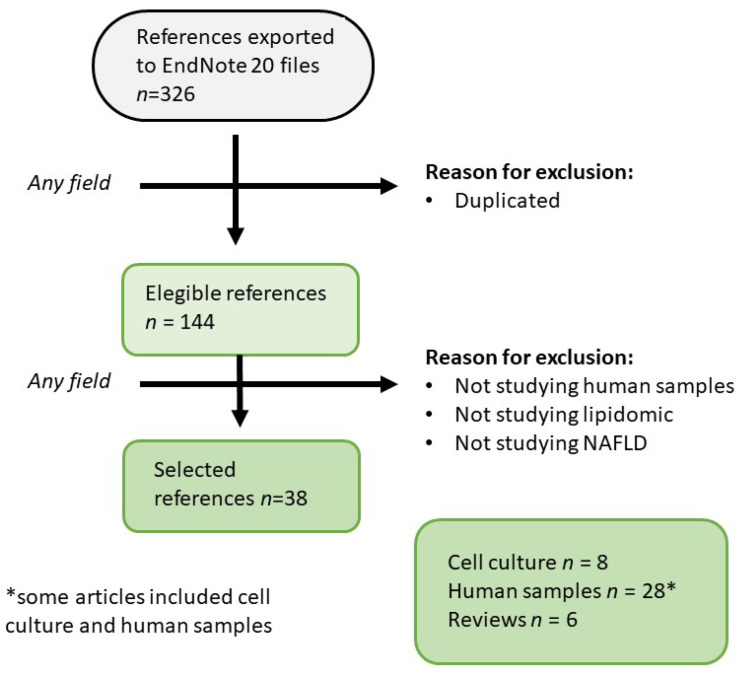
Flow chart displaying the stages used to select the references considered. Endnote 20 (Bld 9325 Thomson Reuters: New York, NY, USA, 2020).

The present review adheres to systematic review guidelines [26], and the keywords used to search PubMed [27] are outlined in Table 1. It provides an updated overview of lipoprotein lipidome studies focusing on NAFLD-related changes in human samples. A total of 326 relevant publications from January 2006 to 2 July 2024 were initially identified. Following the removal of duplicate documents, 144 unique papers were critically evaluated to ensure they analyzed human lipoproteins via lipid mass spectrometry in samples with steatosis (Figure 2). Papers failing to meet these criteria were excluded. Consequently, this review encompasses 38 papers investigating the effects of NAFLD on the human lipoprotein lipidome.

## 2. Human Cell Culture

Human cell culture is a widely used research model in the NAFLD field due to its several advantages. This technique allows a high environmental control with reduced economic cost and lesser biological risk than working with human samples. Using the HuH7 hepatoma cell line, established from hepatoma tissue, two different manuscripts studied the effect of stable transmembrane 6 superfamily member 2 (TM6SF2) silencing. TM6SF2 is a gene whose function is associated with hepatic lipid accumulation and reduction in TG secretion, but the details of its mechanisms remained unknown. Knock-down of TM6SF2 results in intracellular accumulation of TGs, CEs, phosphatidylcholine (PC), and phosphatidylethanolamine (PE). In all of these lipid classes, polyunsaturated lipid species were significantly reduced while saturated and monounsaturated species increased their proportions. The PCs were arachidonic acid (AA, FA 20:4n-6) depleted, and AA was also reduced in the total cellular fatty acid (FA) pool. Synthesis and turnover of the hepatocellular glycerolipids were enhanced. The TM6SF2 knock-down cells secreted lipoprotein-like particles with a smaller diameter than controls, and more lysosome/endosome structures appeared in the knock-down cells. The mitochondrial capacity for palmitate oxidation was also significantly reduced [28]. These results were confirmed by Lukkonen et al. [9] who observed through the same intervention that cells reduced the incorporation of polyunsaturated FA (PUFA) into TGs and PCs, meanwhile the incorporation of FA 16:0 and FA 18:1n-9 into TG, PC, and CE was increased. These observations provide new clues to TM6SF2 function as the changes in membrane lipid composition and dynamics caused by its deficiency disrupt hepatic TG secretion and reproduce in vitro the findings observed in humans carrying the E167K variant of *TM6SF2* [29]. The former authors also used gene silencing methods to characterize the impacts of the depletion of the lipoprotein lipase inhibitor, angiopoietin-like protein 3 (ANGPTL3), on the immortalized human hepatocyte (IHH) lipidome. ANGPTL3-depleted IHH displayed changes in several lipid class compositions characterized by abundant n-6 and n-3 PUFA. This PUFA increase coincided with an elevation of lipid mediators (RvD6, PGD_2_, PGF_2a_, TxB_2_, 10S, 17S-diHDPA, MaR2, 13,14-dehydro,15-oxo-LXA_4_, LTB_4_, 22-OH-MaR1, RvD5_n-3 DPA_, 10S, 17S-diHDHA, 5S, 12S-diHETE, PGE_2_, 4S, 14S-diHDHA) that provide protection from lipotoxic and hypoxia-induced ER stress, hepatic steatosis, and IR or for the recovery from cardiovascular events. CEs were markedly reduced too [28]. Burks et al. [30] also conducted experiments on the silencing of the ANGPTL3 gene in HepG2 cells, observing a reduction in polyunsaturated TG levels without changes in total neutral lipids. Overall, these results highlight the potential application of ANGPTL3 inhibitors in treating NAFLD, given their role in activating intravascular lipolysis [31].

This latter cell line was also used by Matilainen et al. to study in cell culture the effects of orotic acid (OA), an NAFLD inductor in rats. The cells were exposed to 500 μM OA during 5 days observing that OA possibly promotes the first stage of de novo lipogenesis (DNL). However, it does not cause a fully lipogenic transformation in HepG2 cells since the FA 16:0 was decreased and relatively high proportions of FA 16:1n-7 were observed, suggesting an active delta9-desaturation that may limit lipogenesis and the accumulation of toxic FA 16:0. In addition, the observed increase production of long-chain n-3 PUFA and the active incorporation of certain FAs, including FA 18:1n-9, into cells could reduce the inflammatory signaling and the increased proportions of FA 20:4n-6. This introduces the possibility that human hepatocytes may respond to orotic acid by developing fatty liver in a manner similar to rats, yet distinct from other species such as other rodents, chickens, rabbits, pigs, or monkeys [32]. In a more physiological and translational setup, Sazaki et al. [33] studied the effects of the incubation of human liver-derived C3A cell line with oxidized low-density lipoproteins (oxLDLs) and native LDL (nLDL) on their lipid metabolism, lipid droplet formation, and gene expression. Their results showed that nLDL induced lipid droplets enriched with CEs, promoted triglyceride hydrolysis, and inhibited oxidative degeneration of CEs in association with the altered expression of *LIPE*, *FASN*, *SCD1*, *ATGL*, and *CAT* genes. In contrast, oxLDL showed a striking increase in lipid droplets enriched with CE hydroperoxides (CE-OOH) in association with the altered expression of *SREBP1*, *FASN*, and *DGAT1*. Additionally, phosphatidylcholine hydroperoxides (PC-OOH)/PC were also increased in lipid droplets of oxLDL-supplemented cells. According to their observations, they proposed oxLDL as a novel therapeutic target and a candidate biomarker for NAFLD and NASH.

The work with stable cell lines has its own limitations since these usually accumulate numerous mutations leading to the loss of many biotransformation activities [34]. In order to promote the use of iPSC (induced pluripotent stem cells) as a hepatocyte model, Kiamehr et al. [35] described the lipid profile and FA metabolism of hepatocyte-like cells (HLCs) induced by five commonly used methods from three iPSC (induced pluripotent stem cells) lines comparing them with the current standard hepatocyte models: HepG2 cells and primary human hepatocytes (PHHs). They observed that HLCs resembled PHHs in their lipid profile, unlike HepG2 cells. Furthermore, HLCs were able to efficiently use the exogenous FAs available in their medium and simultaneously modify simple lipids into more complex ones to fulfill their needs. The authors concluded that HLCs provide a functional and relevant model to investigate human lipid homeostasis at both molecular and cellular levels.

Accumulated mutations are not the only cell-line culture limitation; these models have been historically limited to unique cell-type models without considering the milieu of stimulus led by the paracrine communications of neighbor cells. This is reflected in the absence of a realistic translational preclinical human model for NASH drug development. To fill this gap, Feaver et al. [36] propose a more realistic model using multicellular approaches. They engineered an in vitro liver model co-culturing primary human hepatocytes, hepatic stellate cells (HSCs), and macrophages. Then, they evaluated the response under a 10-day incubation with a combination of different toxic stress factors (high glucose concentration (25 μM), insulin (6.9 nM), and non-esterified fatty acids (NEFAs): oleic acid (65 μM) + palmitic acid (45 μM)) comparing the outcome with clinical data from NAFLD and NASH patient biopsies. The lipotoxic milieu promoted hepatocyte lipid accumulation (4-fold increase, *p* < 0.01) and a lipidomics signature that was more similar to NASH biopsies than to NAFLD biopsies, characterized by an increase in total lipids due to increases in TGs, DGs, SFAs, NEFAs, CEs, and the n-6/n-3 ratio. Despite the development of this highly physiological model, the manuscript did not scope a comparison with a simple cell culture model to detect the limitations of the simplified hepatocytes culture version while comparing the outcome to clinical data, restraining the significance of the analysis, nor did it include a list of studied lipid species.

These interesting results are summarized in Table 2 and highlight the noteworthiness of cell culture lipid metabolism and lipidomics in NAFLD research. Nevertheless, to our knowledge, no manuscript has addressed a lipidomic perspective of cell culture lipoproteins, and this approach remains to be explored.

## 3. Tissues and Solid Biopsies

Manuscripts including lipidomic approach in solid biopsies to study NAFLD are summarized in Table 3. Human biopsies provide the most valuable source of information for comprehending biological processes involving diverse organs and tissues. This reasoning led Kolak et al. [37] to study subcutaneous adipose tissue of 20 non-diabetic, healthy, obese women divided into normal and high liver fat (LFAT) groups. The study of 154 lipid species revealed several differences between the groups, where the most striking alteration was the increase in TGs in the high LFAT group, particularly those enriched in long-chain FAs (LCFAs), and ceramides (Cer), specifically Cer(d18:1/24:1). This increase in Cer (or their metabolites) could contribute to adverse effects of long-chain fatty acids on IR and inflammation. The same reasoning led Puri and colleagues [38] to study liver biopsies from nine NAFLD, nine NASH patients, and nine healthy donors; they observed that the TGs and DGs increased, PCs decreased and NEFA remained unaltered in NAFLD and NASH patients’ samples. Moreover, they described a stepwise increase in TG/DG and FC/PC ratios from normal livers to NAFL to NASH (38). Moving forward, Scupakova et al. [39] characterized the distribution of specific lipid species in frozen liver biopsies from 23 obese subjects with different degrees of NAFLD severity (grade 0 no steatosis, n = 7; grade 1, n = 4; grade 2, n = 9; and grade 3, n = 3) by performing label-free molecular analysis by MALDI-MSI. This approach allowed the researchers to bypass the limitations of traditional lipidomics analysis using liver homogenates or plasma and to explore lipid composition taking into consideration spatial information and lipid distribution. Spatially resolved lipid profiles showed pronounced differences between non-steatotic and steatotic tissues. Lipid identification allowed them to detect 39 species enriched in non-steatotic tissues and 47 in the steatotic regions, with the TOP 10 non-steatotic: PG(18:2/22:6), PG(18:1/22:6), PS(18:0/22:6), PI(18:0/18:2), PI(16:0/18:2), PG(18:0/22:6), PI(16:0/20:4), PI(17:0/18:2), PI(16:0/22:6), and PI(17:0/20:4); and the TOP 10 steatotic: PE(16:0/18:1), PE(18:0/22:4) or PE(20:0/20:4), PE(18:0/18:1) or DMPE(16:0/18:1), PG(18:1/20:4) or PG(18:2/20:3), PG(18:2/20:4), PA(16:0/18:1), PG(18:1/20:3), PE(16:0/18:2), PG(18:1/18:1), and PE(20:4/16:0). An ulterior network analysis revealed phosphatidyl inositols and AA metabolism as characteristic of non-steatotic regions, whereas LDL and VLDL synthesis was associated with steatotic tissue. The authors conclude that the lipid composition of steatotic and non-steatotic tissue is highly distinct, implying that lipid spatial distribution beyond liver zonation is important for understanding the mechanisms of lipid accumulation in NAFLD.

In addition to nutritional imbalance (main NAFLD etiology), different etiologies of NAFLD coexist nowadays [2,19]. In fact, genetic mutations or infectious diseases can also lead to the development of hepatic steatosis [5]. The former case is the one presented by the carriers of the transmembrane 6 superfamily member 2 E167K gene variant (TM6SF2(EK/KK)) who have an increased risk of NAFLD and NASH, but unlike common ‘obese/metabolic’ NAFLD, these subjects lack hypertriglyceridemia and have a lower risk of cardiovascular disease. In order to characterize the underlying liver lipidome, biopsies were taken from subjects with both TM6SF2 genotypes: mutation carriers (EK/KK) or non-carriers (EE). Liver TGs were higher and liver PCs lower in EK/KK due to an increase in TG species with 48–54 carbons and 0–3 double bonds, also a decrease in TGs containing PUFA was observed. Hepatic CE was 20% higher in EK/KK due to the influence of CE(16:0). PCs were lower in EK/KK, with the polyunsaturated PCs being responsible for the decrease. Regarding NEFAs, a decrease was observed in the EK/KK samples, with FA 16:0, FA 18:0, FA 18:1, and FA 18:2 being significantly lowered too. Nonetheless, hepatic NEFAs were relatively enriched in PUFA leading to the idea that hepatic TG and PC synthesis from PUFA is impaired in TM6SF2 mutation carriers [9]. These results serve as a memorandum that phenotype, liver steatosis, in this instance, emerges from the interplay between environmental factors and genetic background, with both playing pivotal roles in determining the outcome.

The NAFLD final stage has also been extensively explored by lipidomics analysis, particularly due to the global health concern posed by hepatocellular carcinoma (HCC). With HCC patients exhibiting a 50% mortality rate within two years of diagnosis, there is a pressing need to enhance our understanding of molecular pathogenesis, particularly concerning lipid metabolism. Ismail et al. [40] performed untargeted UPLC MS-QTOF lipidomics from resected human HCC tissues and their paired non-tumor hepatic tissues (n = 46). The lipidomics data yielded 604 identified lipids that were divided into six super classes. All Cer and PGs were significantly decreased in HCC tissues compared to non-tumor hepatic tissues. For PC and PE, only PUFA-PC and PUFA-PE were downregulated. Conversely, only SFAs and saturated SM were found downregulated in HCC tumors (40).

Using a less invasive method to describe cellular lipid changes due to NAFLD, Notarnicola et al. [41] analyzed the fatty acid profile in erythrocyte membranes of patients with NAFLD (n = 101) by the gas chromatography method. The subjects with severe NAFLD showed a significant decrease in the ratio of stearic acid to oleic acid, suggesting that an impairment in liver metabolism can impact the circulating cell membrane structure. Subjects with severe NAFLD also showed an increased ratio of vaccenic acid/palmitoleic acid emphasizing the possibility of exploring the lipidomic profile of erythrocyte membranes in the diagnosis and staging of NAFLD. Another non-invasive approach was carried out by Coleman et al. [42] who studied the lipid content in the feces of metabolic syndrome (MetS) patients. They observed a significant increase in 417 lipid features of the fecal lipidome in MetS. PC showed a strong correlation with serum levels of TNFalpha, TGs, and decreasing HDL cholesterol levels. These results together pave the path toward the use of non-invasive methods such as fecal or erythrocyte lipidomics as a non-invasive screening method for NAFLD.

## 4. Plasma

Plasma stands out as the primary sample source in human studies, making it a cornerstone in NAFLD research, with many studies utilizing plasma characterization to describe lipidomic changes. Interestingly, results from plasma lipidome differ depending on the NAFLD etiology. Whereas Luukkonen et al. [9] observed that the presence of the E167K mutation in the *TM6SF2* gene led to the reduction in long-chain and polyunsaturated TGs and PC in plasma without changes in CE, Seessle et al. [43] observed that hereditary hemochromatosis (HH) patients, despite having more hepatic steatosis, had lower serum PC and higher PE compared to the healthy control group, indicating a shift from PC to PE but higher TGs. Strengthening the hypothesis that altered lipid metabolism in HH increases the susceptibility to NAFLD. Disturbed phospholipid metabolism may represent an important factor in the pathogenesis of hepatic steatosis in HH. Another genetic origin that could lead to the development of NAFLD is single-nucleotide polymorphisms (SNPs) of apolipoprotein C3 (*APOC3*), which plays an important role in lipid metabolism and dyslipidemia [5]. Xu et al. [44] studied 34 biopsy-proven NAFLD patients and reported that *APOC3* SNPs are intimately correlated to serum lipidomics in NAFLD patients, with the SNP rs2070667 being the most lipid-altered, exhibiting downregulatory effect on PUFA-containing TG: TG(54:7), TG(54:8), and TG(56:9) and a predisposition to high-grade lobular inflammation. These findings suggest an inhibitory effect of *APOC3* rs2070667 lowering serum levels of PUFA-containing TG. In considering the findings from these studies, summarized in Table 4, it is not negligible that the NAFLD genetic background can highly impact plasma lipidomics, and careful consideration is warranted when interpreting plasma lipid profiles in NAFLD patients.

Genetic disorders are not the only condition that can lead to hepatic steatosis. As mentioned, this condition can also originate from an infectious disease such as hepatitis C virus (HCV), which is known to enhance its replication by co-opting the host liver lipid metabolism. HCV cycle infection requires components related to VLDL assembly; therefore, this virus co-opts the VLDL secretory pathway for its own secretion [45,46,47]. Sheridan et al. [48] studied samples from patients with hepatitis C virus genotypes 3 (HCV-G3) or G1 (HCV-G1), observing an increase in hepatic steatosis in the HCV-G3 group. These authors combined several lipidomic approaches: standard enzymatic methods, GC-MS, and LC-MS, and observed that HCV-G3 patients had decreased serum APOB, lower LDL cholesterol levels, and decreased serum levels of lathosterol, without significant reductions in desmosterol, whereas CEs were paradoxically increased in HCV-G3. Lipidomic analysis also showed that PC(36:3), PC(38:3), PC(36:5), and PC(38:5) were increased in HCV-G1. HCV-G3 infection is an independent risk factor for hepatocellular carcinoma and evidence suggests lipogenic proteins are involved in hepatocarcinogenesis probably in relation to the suppression of cholesterol synthesis via lathosterol. Additionally, HCV therapy with direct-acting antiviral (DAA) also modifies patients’ lipidemia and metabolic status as Casas-Deza et al. [49] observed by NMR analysis. In their study, IR correlated with TGs, but not with LDL or HDL cholesterol following DAA therapy, and significant reductions in IR (−22%) and HDL-TG (−18%) were noted after one year. NAFLD may also originate from alterations in other organs, such as adipose tissue [5,50], muscle [2], or suprarenal glands. The later etiology requires further consideration since Van der Heijden’s findings associated aldosterone and renin levels with hepatic steatosis in the 300-OB cohort study [51]. These results raise an intriguing question: are plasma lipids always the most informative indicators of liver fat infiltration status? Under certain circumstances, other metabolites may prove to be more sensitive markers of NAFLD. Unfortunately, this manuscript does not address this question, as it did not investigate the relationship between plasma lipid parameters and NAFLD.

Hepatic steatosis, due directly to a nutritional imbalance, has received the majority of scientific attention and many papers have been published in this regard. Among the earliest investigations into the plasma lipidome of patients with NAFLD is the study conducted by Puri et al. [52]. They explored plasma from NAFLD (n = 25) and NASH (n = 50) subjects and compared them with lean normal controls (n = 50), observing an increase in MUFA and the hydroxyeicosatetraenoic acids (HETEs; AA-derived oxylipins) and a decrease in PUFA in NAFLD; the increase in HETEs was consistent in NASH samples including a decrease in total plasmalogens lead by dm16:0 and dm18:1n9 [45,52,53]. Later on, these results were confirmed by further researchers. Jurado-Fasoli et al. [54] studied the relationship between steatosis and plasma levels of omega-6 FA (FA 18:2n-6, FA 20:3n-6, FA 20:4n-6, and FA 24:4n-6), omega-3 FA (FA 18:3n-3, FA 20:5n-3, and FA 22:6n-3), and their derived oxylipins through targeted lipidomics in a population of 72 middle-aged adults. The authors observed that plasma levels of omega-6 fatty acids and derived oxylipins, the HETE and dihydroxy-eicosatrienoic acids (DiHETrEs; AA-derived oxylipins), were positively associated with impaired liver function parameters, and with the fatty liver index, in agreement with previous publications [45,52,53]. In addition, individuals with higher omega-6/omega-3 FA and oxylipin ratio showed higher levels of HOMA, total cholesterol, LDL-cholesterol, TGs, and GGT, as well as lower levels of HDL cholesterol. In conclusion, they observed that the omega-6/omega-3 FA and oxylipin ratio, as well as specific omega-6 and omega-3 oxylipin plasma levels, reflects an impaired liver function in middle-aged adults. These results highlight the importance of exploring typically overlooked yet bioactive lipids, such as oxylipins, which may play a critical role in determining the progression of liver steatosis.

Also exploring the FA profile, but this time from plasma phospholipids (plasma FA-PL), Imamura et al. [55] established a FA-pattern score composed of 27 fatty acids that had an inverse association with the likelihood of having hepatic steatosis in the two cohorts of the study (EPIC-InterAct (n = 15,919) and NHANES (n = 1566)). This FA-pattern score was partly characterized by high concentrations of linoleic acid, stearic acid, odd-chain fatty acids, and very long-chain saturated fatty acids and low concentrations of gamma-linolenic acid, palmitic acid, and long-chain monounsaturated fatty acids. Further changed patterns of NEFA and FA-PL were confirmed by Mocciaro et al. [56], who used LC-MS to determine the whole serum lipidomic profile in 89 biopsy-proven NAFLD patients and 20 sex- and age-matched controls. They reported a depletion in PUFA-PL and PUFA-NEFA that could imply a possible defect in the transfer of PUFA from peripheral tissues to the liver in NAFLD. Reduced PUFA liver uptake can stimulate the DNL machinery through the activation of the LXR, SREBP-1, ChREBP, and inhibition of PPARa [57,58,59], adding a new layer of complexity to the FA “spill-over” contribution to NAFLD, where the circulating high NEFA levels are decreased in PUFA and, consequently, stimulate DNL. This mechanism is complementary to the IR activation of DNL, with both being reasons for the accumulation of hepatic and lipoprotein fat in NAFLD [5,45,60]. These two manuscripts together highlight a significant relevance of FA saturation both in PL and NEFA that could be extended (or not) to other lipid groups, with both possibilities being important to understanding NAFLD plasma lipidome composition.

Interestingly, Ismail et al. [40] obtained different results when studying the NAFLD plasma lipidome. These authors explored different stages of NAFLD (HCC subjects (n = 23), chronic liver disease (CLD) (n = 15), and healthy control (n = 15)) by untargeted UPLC MS-QTOF lipidomics. Comparing CLD patients to healthy control donors, TGs were found as the most significantly upregulated lipid class followed by PC and plasmalogens. In contrast, almost all blood lipids were significantly downregulated in HCC patients compared to CLD patients. Cer were found as the most significantly decreased followed by PG, PC, and plasmalogens. Regardless of these major differences, there were also common trends in the transitions between healthy controls, and CLD and HCC patients. In blood, several mostly saturated TGs showed a continued increase in the trajectory toward HCC, accompanied by reduced levels of saturated free fatty acids and saturated LPC [40]. Although not replicating the findings observed in previous studies (as summarized in Table 4), this research sheds light on the distinct influence of HCC tumors on circulating lipids. The lack of reproducibility with prior studies may stem from differences in disease staging; while previous investigations focused on NAFLD, Ismail et al. also considered HCC and CLD, which may originate from nutritional imbalances or other diseases [40].

**Table 4:** Summary of plasma lipidomics results.

References	Sample Size, Sex (% Women), and Average Age	Diagnostic Method	Comparison	Lipidomic Approach (Number of Lipid Molecules)	Lipidomic Features
Seessle et al. (2020) [43]	(n = 74) 33% 50 years	Elastography	Hereditary hemochromatosis (HH) vs. healthy	LC-MS (n.a.)	HH → ↓ PC and ↑ PE and TG No differences were seen for HDL, LDL cholesterol, and total cholesterol
Xu et al. (2020) [44]	(n = 34) 44% 41 years	Biopsy	Polymorphisms of APOC3	LC-MS (19)	SNP rs2070667-A variant → ↓ TG containing PUFA: TG(54:7), TG(54:8), and TG(56:9)
Sheridan et al. (2022) [48]	(n = 112) 28% 48 years	H-MRS	HCV-G3 vs. HCV-G1	LC-MS (n.a.)	HCV-G3 → ↑ hepatic steatosis, ↓ LDL cholesterol, ↓ CE and ↓ lathosterol Both conditions ≈ level desmosterol HCV-G1 → ↑ PC(36:3), PC (38:3), PC(36:5) and PC(38:5)
Van der Heijden et al. (2020) [51]	(n = 302) - % - years	H-MRS	Levels of aldosterone and renin	NMR and LC-MS (231)	Aldosterone and renin associated with steatosis Aldosterone, but not renin, was associated with triglyceridemia Aldosterone was associated with VLDLs
Puri et al. (2009) [52]	(n = 125) - % - years	Biopsy proven (NAFLD and NASH) *	NAFLD and NASH vs. healthy	TLC and GS-MS (266)	NAFLD → ↑ MUFAs and HETEs, ↓ PUFA NASH → ↑ HETEs, ↓ PG
Jurado-Fasoli et al. (2023) [54]	(n = 72) 54% 54 years	Fatty liver index (blood biochemistry parameters)	Levels of n-6 FA, n-3 FA, and their derived oxylipins	LC-MS/MS (79)	n-6 FA and derived oxylipins, HETEs, and DiHETrEs positively correlated with liver function parameters ↑ omega-6/omega-3 fatty acid and oxylipin ratio → ↑ total cholesterol, LDL-c, TG, and GGT and ↓ HDL-c
Imamura et al. (2017) [55]	(n = 27,296) - % - years	ALT plasma levels	FA pattern score ([↑↑] of FA_18:2, FA_18:0, OC-FA, and VLC-SFA and [↓↓] of FA_18:3, FA_16:0, and LC-MUFA)	GS-MS (27)	FA-pattern score associated with lower incidence of T2D FA-pattern score inverse association with the likelihood of having NAFLD
Mocciaro et al. (2023) [56]	(n = 109) 43% 55 years	Biopsy *	NAFLD spectrum vs. controls	LC-MS (276)	NAFLD → ↓ PUFA-PL and PUFA-NEFA
Ismail et al. (2020) [40]	(n = 53) 28% 44 years	Biopsy	HCC and CLD vs. healthy	LC-MS (604)	CLD → ↑ TG, ↑ PC and ↑ plasmalogens HCC → ↓ Almost all blood lipids (PC, PG, Cer, LPE, NEFA and plasmalogens)
McGlinchey et al. (2022) [61]	(n = 627) 46% 52 years	Biopsy	Steatosis vs. NASH vs. fibrosis	LC-MS and GC-MS (176)	15 metabolites unique to steatosis: CE(18:0), Cer(d18:1/23:0), Cer(d18:1/24:0), PC(36:3), PC(38:3), PC(40:4), PC(40:8), TG(51:2), FA 16:0, TG(O-52:2) or TG(P-52:1), 3-OH-benzoic acid, 5-OH-1H-indole-3-acetic acid, indole-3-latic acid, lactic acid and tyrosine 18 metabolites unique to NASH: PC(16:0e/18:1(9Z)), PC(O-32:0), PC(O-32:1), PC(O-36:3), PC(O-38:4), PC(O-38:5), PE(O-38:5) or PE(P-38:4), SM(d34:1), SM(d42:2), TG(18:2/18:1/16:0), TG(49:2), TG(50:0), TG(52:5), TG(53:2), TG(53:4), TG(54:3), TG(54:4), TG(54:6) 15 metabolites unique to fibrosis: PC(32:1), PC(35:4), PC(37:4), PC(40:5), PC(40:6), SM(d36:1), SM(d36:2), SM(d38:2), FA 18:1, 2-OH-butanoic acid, 3-OH-butanoic acid, cholesterol, citric acid, isoleucine and lysine
Mouskeftara (2024) [62]	(n = 37) 40% 54 years	Elastography	NASH vs. healthy	LC-MS/MS (359)	Useful lipidomics changes to construct predictive models: NASH → ↑ DG(16:1/18:0), DG(18:0/18:1), DG(18:1/18:1), DG(18:1/18:2), PC(16:0/16:1), PC(18:0/18:1), PC(18:0/22:5), PI(16:0/20:4), PI(16:1/18:1), LPE(18:0), FA (12:0), FA(18:3w3), CAR (4:0) and ↓ CE (20:4), FA 20:4ω6 or FA 20:5ω3, LPC(20:4), LPC(O-16:1).
			NAFLD vs. healthy and NAFLD vs. NASH	LC-MS/MS (359)	No lipidomic change was useful to construct predictive models.
Velenosi et al. (2022) [63]	(n = 47) 51% 47 years	Elastography and biopsy	Postprandial response in NAFLD vs. healthy	LC-MS and-MS/MS (3469)	Postprandial ↑ of DG in NAFLD but not in controls, dissociated from NAFLD severity and obesity. Postprandial ↑ DG correlates with postprandial insulin levels

* NAFLD has been screened out in healthy volunteers by plasma biochemistry parameters. ↓: reduced; ↑: increased, [↑↑]: high concentrations; [↓↓]: low concentrations; n.a., not available.

To elucidate these differences, McGlinchey et al. [61] developed a metabolomic map across the NAFLD spectrum, defining interconnected metabolic signatures of steatosis (non-alcoholic fatty liver, NASH, and fibrosis) showing specific lipid and metabolite profiles for each stage. They performed LC-MS in serum samples from the European NAFLD Registry patients, representing the full spectrum of NAFLD (n = 627). Using various univariate, multivariate, and machine learning statistical approaches, they were able to identify 15 metabolites unique to steatosis (CE(18:0), Cer(d18:1/23:0), Cer(d18:1/24:0), PC(36:3), PC(38:3), PC(40:4), PC(40:8), TG(51:2), FA 16:0, TG(O-52:2) or TG(P-52:1), 3-OH-benzoic acid, 5-OH-1H-indole-3-acetic acid, indole-3-lactic acid, lactic acid and tyrosine), 18 metabolites associated with NASH (PC(16:0e/18:1(9Z)), PC(O-32:0), PC(O-32:1), PC(O-36:3), PC(O-38:4), PC(O-38:5), PE(O-38:5) or PE(P-38:4), SM(d34:1), SM(d42:2), TG(18:2/18:1/16:0), TG(49:2), TG(50:0), TG(52:5), TG(53:2), TG(53:4), TG(54:3), TG(54:4), TG(54:6), and 15 metabolites linked to fibrosis (PC(32:1), PC(35:4), PC(37:4), PC(40:5), PC(40:6), SM(d36:1), SM(d36:2), SM(d38:2], 2-OH-butanoic acid, 3-OH-butanoic acid, cholesterol, citric acid, isoleucine, lysine, oleic acid). In addition, they were able to detect a key pathophysiological transition point in the progression from fibrosis stage F2–F3 characterized by a decrease in LPC(18:0), LPC(18:1), LPC(18:2), LPC(20:3), LPC(20:4), LPC(22:6), PC(18:0p/18:1(9Z)), PC(35:4), PC(O-34:2), PC(O-34:3) PC(O-36:3), PC(O-36:4), PC(O-36:5), PC(O-38:5), SM(d18:1/24:0), SM(d36:1), SM(d36:2), SM(d38:2), and SM(d41:1), pointing to the importance of metabolic stressors [61]. The use of machine learning has also been proposed more recently by Mouskeftara et al. [62] They constructed a predictive model using machine learning that combined lipidomic data (highlighting the most relevant lipid species changes in Table 4) and biochemical parameters for predicting NASH. However, they were unable to develop a similar predictive model for NAFLD, likely due to the small sample size of just 37 samples in total.

Hepatic energy metabolism is a dynamic process modulated by multiple stimuli. In NAFLD, human studies have typically been focused on the static fasting state. Nevertheless, Velenosi et al. [63] proposed an original perspective of NAFLD metabolism by studying postprandial alterations in hepatic lipid metabolism. A total of 37 patients with NAFLD and 10 healthy control subjects ingested a standardized liquid meal with pre- and postprandial blood sampling. Plasmas were characterized by untargeted lipidomics and a specific increase in three plasma DG species [DG(36:3), DG(36:4), DG(36:5)] was observed postprandially in patients with NAFLD but not in the controls. The increase in plasma DG appears independently of NAFLD severity and obesity, and correlates with postprandial insulin levels. With further experiments, they concluded that this unique feature of NAFLD patients reflects the hepatic secretion of endogenous DGs, rather than meal-derived lipids. DGs are known to be lipotoxic and associated with atherosclerosis, highlighting the importance of extending NAFLD research beyond the fasting state [63]. In line with this manuscript, an enlightening review considering the effect of diet lipid supplementation and plasma lipidemia with special attention to phospholipids is comprised elsewhere. They observed that SFA may increase ceramide concentrations in plasma, triglyceride-rich lipoproteins, and HDLs. Also, milk polar lipids may decrease some molecular species of SM and Cer in plasma and intestine-derived chylomicrons [64]. Altogether, dietary FA and lipid species can stimulate the liver in a specific manner, showing different results depending on the physiological status of the organ, with this phenomenon being reflected in circulating lipids and lipoproteins.

## 5. Lipoproteins

Several lipidomic studies have focused on plasma samples with scarce consideration given to lipoprotein metabolism. As above mentioned, lipoproteins serve as the main carriers of plasma lipids, forming a complex interacting network with distinct classes, coexisting with different physiological roles. Initially, four different classes of lipoproteins were described (ordered by density): CM, VLDLs, LDLs, and HDLs, but nowadays many subclasses and new lipoproteins, such as lp(a), have been described, reflecting the heavy complex metabolism in which they are involved [23,53].

The first work describing the lipidomic composition of lipoproteins, to our knowledge, was carried out by Wiesner and colleagues [65]. They explored cholesterol (FC and CE) and PL (PC, PE, LPC, SM, Cer, and PE-pl) lipoprotein composition of 21 healthy donor plasmas by combining FPLC lipoprotein separation and mass spectrometry. They observed that 60% of PC and 40% of SM were found in HDLs, whereas LDLs carried 50% of lipoprotein SM and 60% of Cer, respectively. Moreover, the HDL fraction contained 60% of PE and PE-pl. In general terms, HDL showed a PL-to-cholesterol ratio of 1.09, VLDLs of 0.64, and LDLs of 0.35. And, when comparing the PL classes, PC was the most abundant phospholipid in all lipoprotein fractions ranging from 65 to 74%. LDLs displayed twice the content of SM (25%) than VLDs or HDLs [66]. Regarding LPC, the authors observed a higher amount in HDL fractions, but they probed that this LPC is associated with albumin since the method they used did not provide a complete separation of albumin and HDL. Regarding the lipid species, analysis of the CE species pattern showed no major differences between lipoprotein fractions while the proportion of PC species showed a higher proportion of polyunsaturated species in HDLs without any variation in the major PC species between the lipoprotein classes. In contrast to PC, LPC showed a pronounced lipoprotein-specific lipid species pattern: LPC(16:0) and LPC(18:0) associated with VLDs and LDLs, and LPC(18:2) was mostly present in HDLs (discarding the albumin interference). The major SM species in all three lipoprotein classes was SM(34:1). The ceramide species pattern (was similar for VLDLs and LDLs, whereas HDLs differed significantly, whereby Cer(16:0) doubled and the proportion of Cer(24:0) reduced 10% in HDLs compared with VLDLs and LDLs. Finally, PE and PE-pl revealed a relative high variation between donors [53,65]. This delineates the lipid composition of lipoproteins in a healthy status, providing a static snapshot devoid of any consideration for pathological or physiological influences.

Fortunately, additional research has examined alterations in the lipoprotein lipidome associated with steatosis and, notwithstanding, genetic conditions that confer protection against steatosis can also impact lipoprotein composition, as demonstrated by Ruhanen et al. [28] through lipidome analysis of lipoproteins of ANGPTL3-LOF human (*n* = 5) and control subjects (*n* = 10). The ANGPTL3 LOF lipoproteins showed a decrease in CEs, LPC, and TGs, with a paradoxical augmentation of the CE species with 16:1 and 18:1 FAs, PUFA-LPC, and PUFA-TG. The authors also described profound changes in the species profiles of SM and PC in all the lipoprotein fractions. ANGPTL3 LOF homozygous subjects contained relatively more long SM species, especially SM(24:1) and SM(24:2), and less short saturated SM. Additionally, they were enriched in alkyl ether PC. The SM/PC ratio was increased in all lipoprotein fractions, even in LDLs, where the elevation of SM(24:1) and SM(24:2) improves their lifespan by making them less prone to aggregation. When inspecting individual FAs, the most prominent difference was a higher proportion of 18:2n-6 in all lipoproteins of the control subjects. Unfortunately, this study did not consider the lipid liver infiltration of the lipoprotein donors, and their results have not been directly linked to this pathology in this manuscript. Continuing with genetic studies, Luukkonen et al. [67] studied the lipoprotein subclasses and their composition of the I148M (rs738409-G) variant in PNPLA3, with higher liver fat content than non-carriers in a T2DM cohort (n = 643) by NMR profiling. In homozygous carriers, PNPLA3-I148M showed a decreased number of VLDL and LDL particles and increased number HDL compared with non-carriers. VLDL particles were smaller and LDL particles larger. This effect was smaller, albeit still significant in the less obese than in the obese cohort.

As stated in the previous paragraph, NAFLD and T2DM often coexist driving detrimental effects in a synergistic manner. Alfadda et al. [68] aimed to understand the changes in circulating lipid and lipoprotein metabolism in patients with T2DM (n = 434) with or without NAFLD, assessed by transient elastography. They interrogated the lipid profile of serum samples by using high-throughput proton NMR metabolomics. Their analysis revealed a significant positive association between steatosis and concentration of PLs, cholesterol, and TGs in VLDL and LDL subclasses, while HDL subclasses were negatively associated. The advanced step of NAFLD, fibrosis, also showed a significant association with concentrations of lipids, PLs, cholesterol, and TGs in very small VLDL, large, and very large HDL subclasses. They concluded that patients with T2DM with higher steatosis grades have altered plasma lipidome. Increased MUFA, SFA, PL, cholesterol, and TG concentrations of VLDL and LDL subclasses are associated with steatosis in patients with T2DM.

Mocciaro et al. [56] focused their research on characterizing the HDL lipid composition using mass spectrometry across the spectrum of NAFLD, given HDL’s role as a potential conduit for delivering hepatic lipids from peripheral tissues alongside NEFA. To achieve this, they used LC-MS in 89 biopsy-proven NAFLD patients and 20 sex- and age-matched controls. Their study revealed lower levels of PUFA-PL in HDL, negatively correlated with BMI, IR, TGs, and hepatocyte ballooning. The NAFLD group also exhibited higher levels of saturated Cer within HDL, positively correlated with IR and transaminases. These findings suggest a potential impairment in the transfer of PUFA from peripheral tissues to the liver in NAFLD. Further mechanistic studies are required to explore the biological implications of these findings, particularly investigating whether alterations in HDL composition could influence liver metabolism and damage, thereby contributing to NAFLD pathophysiology as in atherosclerosis [66]. Other researchers focused their research on revealing VLDL composition and its link to intrahepatic triglyceride (IHTG) accumulation [69]. Despite a reduced number of samples (12 male donors), they described the relationships between concentrations of Cer, DG, and TG in VLDL and IHTG. VLDL particles were isolated from plasma by ultracentrifugation and their content in TG, DG, and Cer was quantified by mass spectrometry obtaining a molar quantity of 137:3:1, TG:DG:Cer, respectively. Among the 200 species of the TGs identified, the five in highest concentration [TG(14:0/18:1/18:2)(5,4%), TG(16:0/16:1/18:1) (6,4%), TG(16:0/18:2/18:2) (10,2%), TG(16:0/18:1/18:1) (11,3%), TG(16:0/18:1/18:2) (13%)] were as abundant as the remaining 195 TG species. Regarding DGs, a total of 21 species were identified but 5 of them represented 75%: DG(18:2/18:2) (7,5%), DG(16:0/18:1) (9,3%), DG(16:0/18:2) (11,1%), DG(18:1/18:1) (20%), and DG(18:1/18:2) (29,7%). A similar phenomenon was observed for Cer, a total of 21 species were identified, but 5 of them represented 75%: Cer(d18:1/23:0) (11%), Cer(d18:1/22:0) (13.5%), Cer(d18:1/24:1) (21.5%), and Cer(d18:1/24:0) (24%). The VLDL ratio of Cer(d18:1/16:0)/Cer(d18:1/24:0) showed a positive correlation with IHTG. Nonetheless, negative correlations were observed between the VLDL content of TG, DG, and Cer and IHTG, which could be in opposition to other authors’ observations by NMR (68). These disparities could be justified by several reasons: (1) a reduced sample size, (2) different exploration conditions (e.g., healthy vs. diabetic, different IHTG stages), and (3) differences in methodology (mass spectrometry vs. NMR).

## 6. Lipoprotein Lipidomics beyond NAFLD

Lipoprotein lipidomics is an emergent field and it has been useful to other disease research beyond hepatic steatosis. For example, Lemes et al. [70] studied the HDL composition of leprosy patients treated with a multidrug therapy, observing an altered lipid profile of patients before treatment. In studies related to the topic of this review, Mocciaro et al. [71] explored the HDL composition of patients with central obesity and metabolic syndrome observing an impairment of phospholipid metabolism (lower PC and SM concentration in HDL, despite higher PC in total serum). Also, Denimal et al. [72] focused their efforts on the characterization of HDL lipidomic abnormalities, this time in T2DM. They observed that the amounts of PC, SM, and S1P were similar in HDL from T2DM patients and controls. PE was almost doubled in T2DM patients, and strikingly, PE-pl, as well as Cer, was lower in HDL from T2D patients. The cholesterol-to-triglyceride ratio was decreased by half in HDL from T2D patients. Finally, Dan et al. [73] explored VLDLs and CM in obese patients challenged to a standardized meal before and after a sleeve gastrectomy (SG). SG decreased postprandial CM TGs, and the degree of the reduction was higher in patients without T2DM. In addition, patients with T2DM had higher TGs in their VLDLs than those without T2DM independently of the SG.

## 7. Lipoprotein Lipidomics Methods and Related Technical Limitations

Synthesizing the results from lipoprotein lipidomics presented in Table 5, nuclear magnetic resonance (NMR) lipidomics has demonstrated a robust capability for classifying and quantifying various classes and subclasses of lipoproteins. However, NMR generally limits its analysis to lipid classes without providing information on individual lipid species. In contrast, mass spectrometry, particularly when combined with lipoprotein separation techniques such as size exclusion chromatography or ultracentrifugation, fulfills this role more comprehensively. Yet, only two studies [56,69] have incorporated mass spectrometry to analyze isolated lipoproteins in relation to NAFLD, primarily focusing their findings on lipid classes and the saturation of fatty acids. This prompts the question of whether there is a need for a more detailed exploration of lipid species within isolated lipoproteins using mass spectrometry. Further research is necessary to determine the specific roles these lipid species play in the metabolism of NAFLD lipoproteins.

Additionally, the investigation into rare bioactive lipids remains limited, with no studies reporting on glycosphingolipids or oxysterols. This oversight may be attributed to technical challenges; glycosphingolipids typically elute with the aqueous phase during lipid extractions, and oxysterol analysis often requires large sample volumes and specialized techniques. However, examining these lipids could prove crucial, as minor variations in their levels can have significant biological, potentially antagonistic, effects [74,75].

## 8. Statistical Analysis Techniques in Lipidomic Studies

Various manuscripts have employed different statistical tests for analyzing individual lipid species, including the unpaired two-tailed *t* test [29], Mann–Whitney U test [9], Welch’s corrected *t* test [43], one-way ANOVA [33], and the Wilcoxon test [40], tailored to the experimental model and data distribution to derive *p* values. Subsequently, *p* values were adjusted for multiple comparisons using methods such as the Benjamini–Hochberg correction [36,51]. Furthermore, several studies incorporated multivariate analyses to detect significant lipid species and patterns in NAFLD research, including principal component analysis (PCA) [29], partial least squares discriminant analysis (PLS-DA) [28], PCA coupled with linear discriminant analysis (PCA-LDA) [39], sparse partial least squares–discrimination analysis (sPLS–DA) [40], and orthogonal projections to latent structures discriminant analysis (OPLS-DA) [62]. Additionally, some manuscripts explored lipid pathways analysis [30] and utilized machine learning technology to predict NAFLD based on specific plasma lipid species [62]. Integrating these statistical techniques with lipidomic data paves the way for discovering new biomarkers and elucidating novel metabolic pathways.

## 9. Conclusions and Future Perspectives

An important limitation of this review is its narrow focus on lipidomic analysis, excluding a broader metabolic perspective. Integrating data from other omic sciences, such as metabolomics or proteomics, could provide additional insights that were not explored here. While lipoprotein lipidomics is a developing field in the characterization and monitoring of NAFLD, it is still in its early stages. However, the initial findings are promising, offering valuable insights into crucial aspects of the disease.

**Figure 3:**
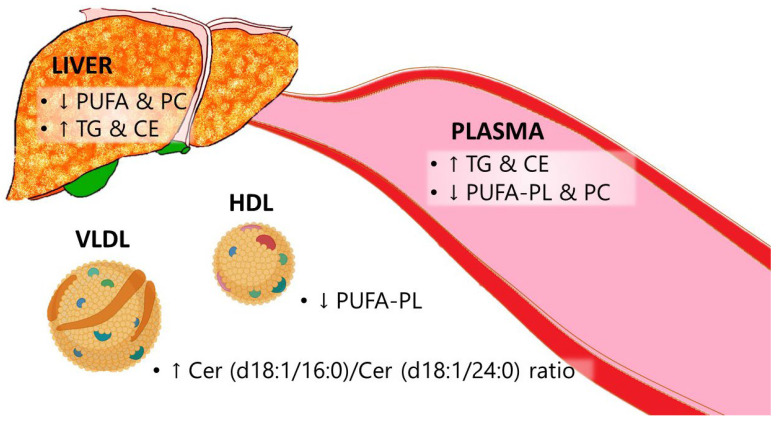
Scheme showing the common lipidomic changes observed by reviewed manuscripts. CE: Cholesterol ester. Cer: Ceramide. HDL: High-density lipoprotein. PC: Phosphatidylcholine. PUFA: Polyunsaturated fatty acid. PUFA-PL: Polyunsaturated fatty acid phospholipids. TG: Triglycerides. VLDL: Very low-density lipoprotein. ↑, increase, and ↓, decrease.

In steatotic conditions, cultured hepatocytes demonstrate decreased incorporation of PUFA into lipids, leading to elevated levels of TGs, CE, and PC. The former feature is also reported in liver biopsies, where increases in TGs and CEs are noted, but most cases show a decrease in PC in steatosis conditions. With the establishment of HCC, a unique and characteristic lipidome emerges, marked by decreases in Cer, PG, PUFA-PC, PUFA-PE, and saturated SM. This reduction in PUFA-PL and PC, along with increases in TGs and CEs (except for the E167K mutation in TM6SF2), is also reflected in plasma samples, as evidenced in lipoprotein lipidomic changes summarized in Figure 3.

Despite these shared characteristics, it is crucial to acknowledge that non-alcoholic steatosis can arise from various etiologies, such as genetic disorders (e.g., TM6SF2, APOC3, HH), infectious agents (e.g., HCV, leprosy), hepatotoxic drugs, and nutritional imbalances, rather than being a singular condition. Given the diverse causes and the variable progression to fibrosis, cirrhosis, and HCC, there continues to be a debate regarding the most fitting nomenclature. Several terms have been proposed, including NAFLD, MAFLD, and the recently accepted MASLD [1].

Furthermore, this review highlights two crucial aspects of sampling. Firstly, there is a growing interest in exploring new non-invasive sample sources such as stool, urine, and saliva, alongside traditional plasma samples. Secondly, it is important to recognize that lipids in plasma are compartmentalized into various lipoproteins, each with distinct compositions and functions. Conducting a detailed analysis of these isolated lipoproteins could enhance our understanding of lipid metabolism and liver health. Such insights are particularly valuable for projects like the NIMBLE (Non-Invasive Biomarkers of Metabolic Liver Disease) consortium, which aims to develop non-invasive diagnostic tools [76].

Finally, further attention should be given to metabolic challenges. It is crucial to stimulate liver metabolism not only through fasting but also by employing different standardized meals enriched with various lipids (TGs, CEs, Cer, PC, PE, etc.). Analyzing the differential response in the postprandial state could represent a significant advance in characterizing NAFLD, particularly as this reflects the most common metabolic state in modern societies. Additionally, given the novelty of the lipidomics approach, particularly in the realm of lipoprotein lipidomics, it is premature to restrict research to a few lipid species. With the decreasing costs of lipidomic analyses and the advancement of AI technologies, limiting the scope to specific lipid species could be counterproductive. As we advance, expanding our investigation to a broader array of lipid species will likely yield more comprehensive insights into the lipidomic contributions to NAFLD.

Considering the limitations of invasive diagnostic methods, the emergence of plasma lipoprotein lipidomics as a surrogate biomarker holds significant promise due to its wealth of information and relative non-invasiveness. Despite these advantages, there remains a considerable journey ahead to establish its critical role in addressing such a complex condition with diverse etiologies.

## Figures and Tables

**Table 1:** Combination of keywords used to search the PubMed database.

Keyword Combinations	Number of References
Lipoprotein + NAFLD + lipidomic	42
HDL + NAFLD + lipidomic	11
LDL + NAFLD + lipidomic	22
VLDL + NAFLD + lipidomic	12
Lipoprotein + steatosis + lipidomic	123
HDL + steatosis + lipidomic	36
LDL + steatosis + lipidomic	50
VLDL + steatosis + lipidomic	27
Lipoprotein + MAFLD + lipidomic	1
HDL + MAFLD + lipidomic	1
LDL + MAFLD + lipidomic	1
VLDL + MAFLD + lipidomic	0

**Table 2:** Summary of lipidomics results from cell culture experiments.

References	Experimental Model	Treatment	Lipidomic Approach (Number of Lipid Molecules)	Lipidomic Features
Luukkonen et al. (2017) [9]	HuH7 hepatome cells	TM6SF KD	LC-MS (186)	↓ PUFA into TG and PC ↑ FA 16:0 and FA 18:1n-9 into TG, PC and CE
Ruhanen et al. (2020) [28]	Immortalized human hepatocytes (IHH)	ANGPTL3 KD	DI-MS/MS and GC-MS (168)	↑ PUFA ↓ CE ↑ lipid mediators (RvD6, PGD_2_, PGF_2a_, TxB_2_, 10S, 17S-diHDPA, MaR2, 13,14-dehydro,15-oxo-LXA_4_, LTB_4_, 22-OH-MaR1, RvD5n-3 DPA, 10S, 17S-diHDHA, 5S, 12S-diHETE, PGE_2_, 4S, 14S-diHDHA)
Ruhanen et al. (2017) [29]	HuH7 hepatome cells	TM6SF KD	DI-MS/MS and GC-MS (144)	Intracellular accumulation of TG, CE, PC, and PE with ↓ PUFA species and ↑ SFA and MUFA species ↓ FA 20:4n-6 in PC
Burks et al. (2024) [30]	HepG2	ANGPTL3 KO	LC-MS (73)	ANGPTL3 −/−: ↓ TG(16:1, 16:1,20:3), TG(16:1, 16:1, 18:2), TG (20:3, 18:2, 16:0), TG(20:3, 18:1, 16:0), TG (16:1, 18:1, 18:2) and TG (16.0, 16:1, 18:2) but no change in total neutral lipid accumulation ANGPTL3 −/−: ↓ PUFA, fatty acids with double bonds, and TG ANGPTL3 −/− and RLDL −/−:
Matilainen et al. (2020) [32]	HepG2	Orotic acid [500 μM] 5 days	GC-MS (45)	↓ FA 16:0, ↑ FA 16:1n-7 and ↑ PUFA (FA 18:1n-9, FA 20:4n-6 and FA 22:6n-3)
Sazaki et al. (2023) [33]	Human liver-derived C3A cell line	incubation with nLDLs or oxLDLs	LC-MS/MS (56)	nLDL → ↑ CE enrich LD and ↑ TG hydrolysis nLDL → ↓ oxidative degeneration of CE oxLDL → ↑ CE hydroperoxides (CE-OOH) enriched LD and ↑ (PC)-OOH/PC ratio
Kiamehr et al. (2019) [35]	Hepatocyte-like cells (HLCs) from iPSC	HLC vs. HepG2 and Primary Human Hepatocytes (PHH)	LC-MS and GC-MS (145)	HLCs resembled PHHs in their lipid and FA profile HLCs contained low levels of lysophospholipids compared to HepG2
Feaver et al. (2016) [36]	In vitro liver model of hepatocytes, HSCs and macrophages	10 days combination of (glucose [24 μM], insulin [6.9 nM], NEFA (OA [65 μM] + PA [45 μM])	LC-MS (767)	↑ Total lipids lead by ↑ TG, DG, MUFA, SFA, NEFA and CE ↑ n-6/n-3 ratio

↓: reduced; ↑: increased; HSC: hepatic stellate cells.

**Table 3:** Summary of tissue and solid biopsy lipidomics results.

References	Tissue	Sample Size, Sex (♀%) and Average Age	Diagnostic Method	Comparison	Lipidomic Approach (Number of Lipid Molecules)	Lipidomic Features
Kolak et al. (2007) [37]	scWAT of obese women	(n = 20) 100% no age	H-MRS	Normal vs. high liver fat (LFAT)	LC-MS (154)	↑ TG in LFAT group, especially those carrying LCFA ↑ Cer in LFAT group, specifically Cer(d18:1/24:1)
Puri et al. (2007) [38]	Liver	(n = 27) 70% 47 years	Biopsy	NAFLD and NASH vs. healthy	TLC and GS-MS (n.a.)	↑ DG and TG, unaltered NEFA and ↓ PC. Stepwise ↑ in TG/DG and FC/PC ratios from normal livers to NAFL to NASH.
Puri et al. (2007) [38]	Liver	(n = 27) 70% 47 years	Biopsy	NASH vs. NAFLD and healthy	TLC and GS-MS (n.a.)	↓ 20:4n-6 in NEFA, TG, and PC. ↓ EPA and DHA in TG. ↑ n-6:n-3 NEFA ratio
Scupakova et al. (2018) [39]	Liver from obese patients	(n = 23) 70% 43 years	Biopsy	Steatotic areas vs. non-steatotic areas	MALDI-MSI (84)	TOP10 non-steatotic area lipid species: PG(18:2/22:6), PG(18:1/22:6), PS (18:0/22:6), PI(18:0/18:2), PI(16:0/18:2), PG(18:0/22:6), PI(16:0/20:4), PI(17:0/18:2), PI(16:0/22:6), PI(17:0/20:4) TOP10 steatotic area lipid species: PE(16:0/18:1), PE(18:0/22:4) or PE(20:0/20:4), PE(18:0/18:1) or DMPE(16:0/18:1), PG(18:1/20:4) or PG(18:2/20:3), PG(18:2/20:4), PA(16:0/18:1), PG(18:1/20:3), PE(16:0/18:2), PG(18:1/18:1), PE(20:4/16:0). ↑ PI and arachidonic acid metabolism in non-steatotic regions LDL and VLDL metabolisms were associated with steatotic tissue
Luukkonen et al. (2017) [9]	Liver	(n = 94) 71% 46 years	Biopsy	EK/KK vs. EE genotype	LC-MS and GC-MS (n.a.)	EK/KK → ↑ TG and CE, ↓ PC and NEFA EK/KK → PUFA ↓ in PC but ↑ in NEFA
Ismail et al. (2020) [40]	Liver	(n = 53) 28% 44 years	Biopsy	Human HCC vs. paired non-tumor hepatic tissue	LC-MS (604)	Almost all lipids (Cer, PC, PG, PE, and plasmalogens) were downregulated in HCC tissues.
Notarnicola et al. (2017) [41]	Erythrocyte membranes	(n = 101) 46% no age	Elastography	NAFLD spectrum vs. controls	GC-MS (n.a.)	↓ FA 18:0/FA 18:1n-9 ratio according to the degree of liver damage ↑ FA 18:1n-7/FA 16:1n-7 ratio in severe NAFLD
Coleman et al. (2022) [42]	Feces	(n = 18) 61% 46 years	-	MetS patients vs. healthy	LC-MS (7453 *)	↑ 417 lipid in MetS patients

↓: reduced; ↑: increased; n.a., not available. * mass spectrometer features.

**Table 5:** Summary of lipoprotein lipidomics results.

References	Sample Size	Diagnostic Method	Comparison	Lipidomic Approach (Number of Lipid Molecules)	Lipidomic Features
Wiesner et al. (2009) [65]	(n = 21)	-	Composition of healthy lipoproteins	FPLC and LC-MS (88)	HDL contained: 60% of PC, PE, and PE-pl and 40% of SM LDL contained: 50% of SM and 60% of Cer. PL to cholesterol ratio of: HDL, 1.09; VLDL, 0.64; and LDL, 0.35 PC: the most abundant PL in all lipoproteins (65–74%). LPC: mainly associated with albumin. CE species pattern showed no major differences between lipoproteins. PC species showed a higher proportion of PUFA species in HDL LPC species are lipoprotein specific: LPC(16:0) and LPC(18:0) associated to VLDs and LDL, and LPC(18:2) mostly present in HDL. The major SM species in all three lipoprotein classes was SM(34:1). PE and PE-pl revealed a relatively high variation between donors
Ruhanen et al. (2020) [28]	(n = 15)	-	ANGPTL3-LOF vs. control lipoproteins	Ultracentrifugation, DI-MS/MS, and GC-MS (168)	↓ TG, LPC, and CE, but ↑ CE with 16:1 and 18:1 FAs and ↑ PUFA-LPC and PUFA-TG ↑ SM_24:1 and SM_24:2 and ↑alkyl ether PC ↑ SM/PC ratio
Luukkonen et al. (2021) [67]	(n = 643)	Biopsy and H-MRS	PNPLA3-I148M variant vs. noncarriers	NMR (n.a.)	↓ VLDL and LDL particles and ↑ HDL Smaller VLDL particles and larger LDL particles no effect in patients with lower IR reduction in the effect in less obese patients
Alfadda et al. (2023) [68]	(n = 434)	Elastography	T2DM patients: NAFLD vs. non-NAFLD lipoproteins	NMR (na)	positive association of concentration of lipids, PL, cholesterol, and TG in VLDL and LDL subclasses with NAFLD negative association between steatosis and HDL subclasses with NAFLD
Mucinski et al. (2020) [69]	(n = 12)	H-MRS	VLDL: Composition and correlation with IHTG	Ultracentrifugation and LC-MS (242)	VLDL molar proportions of TG:DG:Cer(137:3:1) The five most abundant TG represent 50% of the TG in VLDL (TG(14:0/18:1/18:2), TG(16:0/16:1/18:1), TG(16:0/18:2/18:2), TG(16:0/18:1/18:1), and TG(16:0/18:1/18:2)) The five most abundant DGs represent 75% of the DGs in VLDL (DG(18:2/18:2), DG(16:0/18:1), DG(16:0/18:2), DG(18:1/18:1), and DG(18:1/18:2)) The five most abundant Cer represent 75% of the Cer in VLDL (Cer(d18:1/23:0), Cer(d18:1/22:0), Cer(d18:1/24:1), and Cer(d18:1/24:0)) Negative correlations of VLDL content of TG, DG, and Cer and IHTG Ratio of Cer(d18:1/16:0)/Cer(d18:1/24:0) positive correlation with IHTG
Mocciaro et al. (2023) [71]	(n = 109)	Biopsy	HDL: NAFLD spectrum vs. healthy donors	LC-MS (276)	PUFA PL in HDL negatively correlated with BMI, IR, TG, and hepatocyte ballooning NAFLD HDLs → ↑ saturated Cer

↓: reduced; ↑: increased.

## Data Availability

Not applicable.

## Abbreviations

- CE: Cholesterol ester - Cer: Ceramides - CLD: Chronic liver disease - DG: Diglycerides - DiHETrEs: Dihydroxy-eicosatrienoic acids - DNL: De novo lipogenesis - FA: Fatty acid - FC: Free cholesterol - LCFA: Long-chain fatty acid - LC-MS: Liquid chromatography–mass spectrometry - LPC: Lysophosphatidylcholine - LFAT: high-fat liver - HCC: Hepatocellular carcinoma - HH: Hereditary hemochromatosis - HETEs: Hydroxyeicosatetraenoic acids - IHTG: Intrahepatic triglyceride - IR: Insulin Resistance - MALDI-MSI: Matrix-assisted laser desorption/ionization–mass spectrometry imaging - MetS: Metabolic syndrome - MUFA: Monounsaturated fatty acid	- NAFLD: Non-alcoholic fatty liver disease - NEFA: Non-esterified fatty acid - NMR: Nuclear magnetic resonance - PC: Phosphatidylcholine - PE: Phosphatidylethanolamine - PhC: Phosphocholine - PL: Phospholipid - PE-pl: PE-based plasmalogens - PNPLA3: Patatin-like phospholipase domain containing 3 - PUFA: Polyunsaturated fatty acid. - RBC: Red blood cell - SG: Sleeve gastrectomy - SFA: Saturated fatty acid - SM: Sphingomyelin - S1P: Sphingosine 1 phosphate - TG: Triglyceride - TM6SF2: Transmembrane 6 superfamily member 2 - T2DM: Type 2 Diabetes mellitus - UPLC MS-QTOF: Ultraperformance liquid chromatography–Time-of-flight mass spectrometry
